# Do Spatially-Implicit Estimates of Neutral Migration Comply with Seed Dispersal Data in Tropical Forests?

**DOI:** 10.1371/journal.pone.0072497

**Published:** 2013-08-19

**Authors:** François Munoz, Champak R. Beeravolu, Raphaël Pélissier, Pierre Couteron

**Affiliations:** 1 Université Montpellier 2, UMR AMAP, Montpellier, France; 2 IRD, UMR AMAP, Montpellier, France; 3 INRA, UMR CBGP, Montferrier-sur-Lez, France; 4 Institut Français de Pondichéry (IFP), UMIFRE 21 CNRS-MAEE, Puducherry, India; Swiss Federal Institute of Technology (ETH Zurich), Switzerland

## Abstract

Neutral community models have shown that limited migration can have a pervasive influence on the taxonomic composition of local communities even when all individuals are assumed of equivalent ecological fitness. Notably, the spatially implicit neutral theory yields a single parameter *I* for the immigration-drift equilibrium in a local community. In the case of plants, seed dispersal is considered as a defining moment of the immigration process and has attracted empirical and theoretical work. In this paper, we consider a version of the immigration parameter *I* depending on dispersal limitation from the neighbourhood of a community. Seed dispersal distance is alternatively modelled using a distribution that decreases quickly in the tails (thin-tailed Gaussian kernel) and another that enhances the chance of dispersal events over very long distances (heavily fat-tailed Cauchy kernel). Our analysis highlights two contrasting situations, where *I* is either mainly sensitive to community size (related to ecological drift) under the heavily fat-tailed kernel or mainly sensitive to dispersal distance under the thin-tailed kernel. We review dispersal distances of rainforest trees from field studies and assess the consistency between published estimates of *I* based on spatially-implicit models and the predictions of the kernel-based model in tropical forest plots. Most estimates of *I* were derived from large plots (10–50 ha) and were too large to be accounted for by a Cauchy kernel. Conversely, a fraction of the estimates based on multiple smaller plots (1 ha) appeared too small to be consistent with reported ranges of dispersal distances in tropical forests. Very large estimates may reflect within-plot habitat heterogeneity or estimation problems, while the smallest estimates likely imply other factors inhibiting migration beyond dispersal limitation. Our study underscores the need for interpreting *I* as an integrative index of migration limitation which, besides the limited seed dispersal, possibly includes habitat filtering or fragmentation.

## Introduction

Community ecology underwent a sea-change in 2001 with the advent of the neutral theory of biodiversity [1]. In his seminal contribution, Hubbell [2] assumed that species biodiversity at local and regional scales is maintained by a stochastic interplay of birth, death, migration and speciation processes involving individuals of equivalent fitness. He argued that propagule dispersal limitation is a universal and strong enough constraint on migration which generates the complex observed patterns of local species coexistence. The spatially-implicit neutral model (SINM [2], [3]) defines the migration process as the rate at which individuals originating from a regional source pool or metacommunity establish themselves in a local community. A single parameter, *I*, is then used to represent the effective number of immigrants competing with the offspring of a local community to replace a dead local individual (zero-sum dynamics) [4]. Ever since, the scope of *I* has been broadened to account for limits to species movement other than their dispersal abilities such as habitat filtering, physical barriers and anthropogenic fragmentation [5], [6]. The original SINM, along with some of its later variants have been used to infer *I* and thereby the intensity of immigration with respect to the taxonomic composition of the local communities [5], [7]–[9]. A still unresolved issue is to determine to what extent the inferred immigration parameter from field data relates to features of propagule dispersal and to other causes obviating immigration from the regional pool into the local community. In this regard, and given the renewed call for a regional perspective to community ecology [10], [11], disentangling the effects of genuine local dispersal limitation, as initially invoked by Hubbell [2], from the regional processes of species migration is still a central issue.

Investigating the nature of migration limitation is particularly difficult when the system under study is a continuous landscape (e.g., a non-fragmented tropical wet forest), where local communities have no a priori delineation and where the consequences of habitat heterogeneity are not known. On the other hand, as an alternative to the SINMs, spatially-explicit or continuous models (SCNM, [3]) have acknowledged the effect of propagule dispersal by modelling the dynamics of individuals in a suitable and uniform environment, without referring to a regional pool of migrants [12]–[14]. The probability of dispersal success has thus been represented as a decreasing function of the distance from the mother individual which is summarized by a dispersal parameter (generally the mean dispersal distance) and the shape of the distribution, e.g. thin- vs. fat-tailed according to the relative importance of long- vs. short-distance dispersal events [15]. Despite this gain in realism, the existing SCNMs do not acknowledge physical barriers or habitat heterogeneity explicitly, and therefore cannot account for the multiple drivers of limited immigration frequently encountered in a real-world scenario. By focusing on ‘dispersal limitation’ in its spatially-explicit approach and on ‘migration limitation’ in its spatially-implicit formulation [16], the neutral theory here mirrors the complexity of dealing simultaneously with both local and regional processes acting at distinct spatial scales.

In this paper, we explore the extent to which SINM-based estimations of the *I* parameter reflects processes, other than pure seed dispersal, responsible for a limited migration of species. In order to assess the extent to which habitat and physical barriers hinder migration beyond the limited dispersal abilities of species, we need a baseline model as to predict the value of the SINM’s immigration parameter *I* that is expected if dispersal limitation of propagules from parent individuals was the only factor. A first attempt by Chisholm & Lichstein [17] proposed an analytical approximation of *I* under strict dispersal limitation, by modelling the flux of propagules landing in a region of fixed area and shape (e.g. a forest plot) under a well-known seed dispersal kernel. This study remains limited due to the assumption of short dispersal distance with respect to the dimensions of the field plot. Here, we extend their approach by relaxing this assumption while considering both the Gaussian and Cauchy dispersal kernels to represent contrasted thin-tailed and heavily fat-tailed kernels, respectively [15]. We compared the values predicted by our approach to values of the SINM’s *I* parameter that were estimated from the taxonomic composition of tropical forest plots, based on various estimation methods (Table 1). We also made use of a review of field studies [18] (Table 2) and of a large database on forest tree species traits from French Guiana [19] (Figure S1), in order to determine realistic bounds of seed dispersal distances in the context of tropical rainforests. With respect to predictions of *I* from a baseline seed dispersal limitation model, additional causes of migration limitation acting at regional scales (e.g. physical and ecological barriers) are expected to yield *I* estimates substantially below this baseline. On the other hand, excessively high estimates of *I* may point towards weaknesses of the estimation methods or violations of some important assumptions that underline them [8], [20].

**Table 1:** Published estimates of the immigration parameter *I* in rainforest tree communities, using Spatially Implicit Neutral Models (SINMs).

Plot code		*A*	*J_s_*	*S*	*m*	*I*	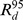 *(Gaussian)*	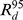 *(Cauchy)*
	**Single large forest plot datasets**							
	*Based on taxonomic diversity alone*							
A	Barro Colorado Island, Panama [44]	50 ha	21457	225	**0.093**	2200	122 m	>10 km
BC	Yasuni National Park, Ecuador [44], [45]	25 ha50 ha	7613 17546	546 821	**0.5** **0.429**	7612 13182	391 m 504 m	>10 km >10 km
D	Korup National Park, Cameroon [44]	50 ha	24591	308	**0.547**	29693	818 m	>10 km
E	Pasoh Forest Reserve, Malaysia [44]	50 ha	26554	678	**0.093**	2722.6	147 m	>10 km
F	Sinharaja, Sri Lanka [44]	25 ha	16936	167	**0.0019**	32.3	3 m	2 m
G	Lambir Hills, Malaysia [44]	52 ha	33175	1004	**0.115**	4310.7	216 m	>10 km
H	Western Ghats, India [46]	30 ha	13383	148	**0.082**	1195.3	86 m	>10 km
	*Including phylogenetic information* [23]							
I	Barro Colorado Island, Panama	50 ha	20788	236	**0.002**	41.66	2.5 m	∼0
J	La Planada, Colombia	25 ha	14100	164	**0.003**	42.42	4 m	55 m
K	Pasoh Forest Reserve, Malaysia	50 ha	29257	674	**0.01**	295.5	18 m	>10 km
L	Lambir Hills, Malaysia	52 ha	29890	990	**0.008**	241.0	14 m	6205 m
	**Multiple plot datasets**							
M	Baro Colarado subplot, Cocoli and Sherman plots, Panama [9]	∼5 ha/plot	1079–2860	99–171		**30.7**–**54.2**	8 m	419 m
N	Western Ghats, India [5]	1 ha/plot	∼400/plt	∼45/plt	0.01–0.11; 0.003–0.08	**4.7**–**50**; **1.3**–**35** ^4^	10 m	436 m
O	Panama Canal Watershed [24]	1 ha/plot	∼400/plt	∼78/plt	**0.05**–**0.3**	21–171	34 m	4334 m

**Figure 1:**
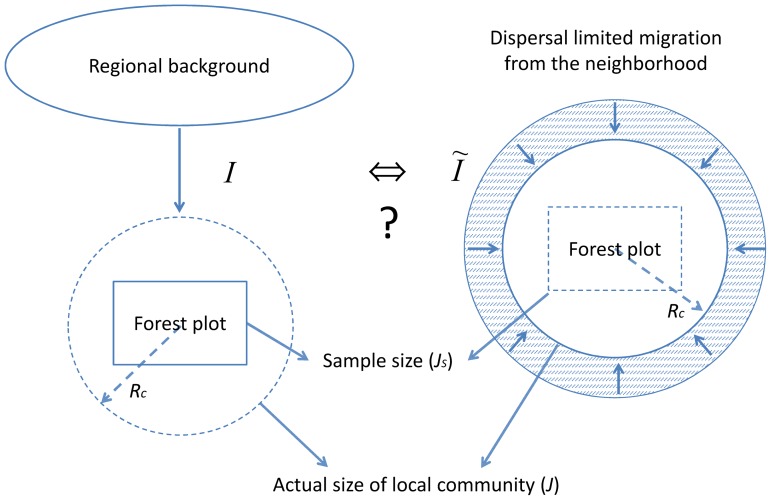
Comparing spatially-implicit immigration from a regional pool to a model based on seed dispersal from the community neighbourhood. A hypothetical rectangular forest plot is shown. In a spatially-implicit framework (left), the plot is part of a discrete local community, which is related to a regional species pool via immigration. Based on the composition of the plot, the SINM based methods allow estimating the number of immigrants available for replacement of a dead individual at the scale of the entire local community. If dispersal limitation is assumed to be the only driver of immigration into the local community (right), the number of incoming individuals from the neighbourhood around the community can be modelled with the help of a dispersal kernel model.

The corresponding 95% quantile dispersal distances, 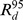, are given under the assumption of pure dispersal limitation using the Gaussian and Cauchy kernels (see main text). *A* is the sample area in ha and *J_s_* corresponds to the sample size in number of individuals above 10 cm dbh. *S* indicates the respective species richness of the sample plots. Bold/normal values respectively denote the published/transformed parameter values of Hubbell's [2] migration rate *m* or the corresponding immigration parameter, *I* [21]. *m*/*I* and 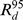 are calculated here for the case when the forest plots represent complete communities (i.e. sample size  =  community size in Figure 1). 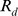 values have been rounded to the nearest metre.

**Table 2:** Some orders of magnitude of seed dispersal distances of tree species in tropical rainforests, as estimated from field studies (extracted from [18]).

Dispersal mode	Species name	Measure of dispersal distance		Site
		mean/median/other	maximum	
Autochory	*Eperua falcata*	60% of trees within 10 m	30 m	French Guiana
Anemochory	*Lophopetalum wightianum*	median at 15–43 m	30–80 m	Western Ghats, India
Anemochory	*Platypodium elegans*	median at 10–23 m	75–105 m	Barro Colorado Island, Panama
Anemochory	*Swintonia schwenkii*	-	few fruits > 50 m	Gunung Gadut, Sumatra
Zoochory (bat)	*Carollia perspicillata*	90% of seeds within 50 m	few seeds > 300 m	Costa Rica
Zoochory (bird)	*various*	mean & median at 100–300 m	-	Rwanda (montane forest)
Zoochory (monkey)	*various*	mean at 76–440 m	288–575 m	La Macarena, Columbia

## Methods

### Spatially-implicit estimation of the immigration number

The neutral spatially-implicit theory is based on the coupling of discrete communities to a regional background via immigration (Figure 1 left). In the initial model of Hubbell [2], the immigration rate, *m*, denotes the probability that a new immigrant replaces a dead resident in the local community. Later publications have introduced the "fundamental immigration number", hereafter called the immigration parameter, *I* [4], [7], [21], which represents the effective number of immigrants competing with the offspring of residents to replace a dead individual in the local community, so that 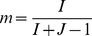, where *J* is the number of residents. As such, *I* embodies the migration-drift equilibrium driving community dynamics, under the assumption that the migrants originate from a far larger spatial scale called the regional background. The spatially-implicit framework is then based on two fundamental assumptions: (i) that the local offspring competing for the replacement of a dead individual are drawn with equal probability from any individual within the local community (panmixia [22]) and (ii) the migrants are drawn from the same regional background for all local communities.

A number of methods have been proposed (most of them based on a coalescent reasoning) to estimate *m* and *I* from community composition on the basis of SINMs. A desirable property of the coalescent-based approaches [5], [7], [23], [24] is that they apply to any sample of *J_s_* individuals drawn from the local community (Figure 1 left). The actual migration-drift balance measured by *I* is directly dependent on community size *J* (that is sample-independence) whereas 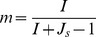 is always sample-dependent (Appendix S5 of [17], Figure 2 of [24]).

**Figure 2:**
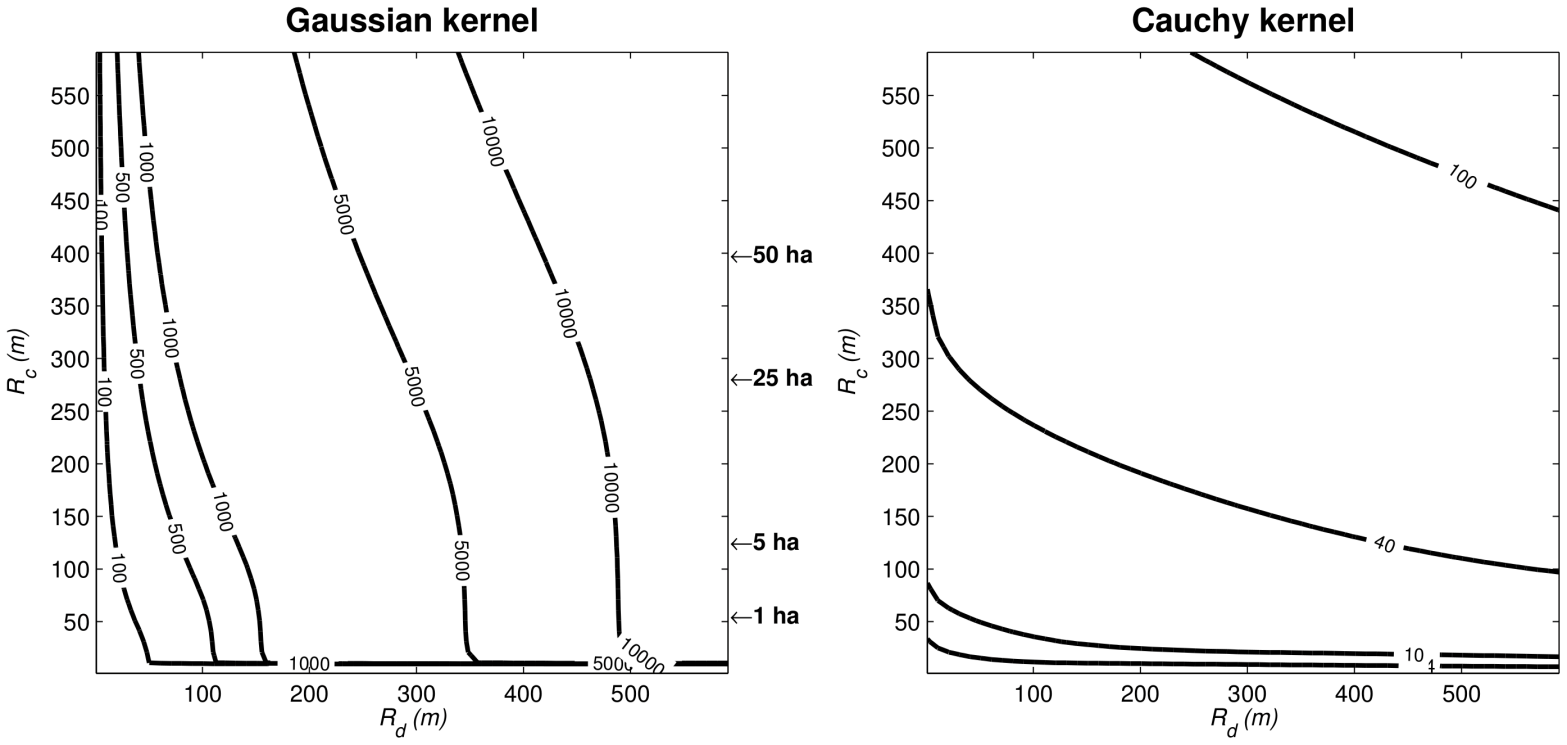
Isolines of the dispersal kernel-based analogue *I˜* of the immigration parameter (Eq. 1) computed as a function of the community radius *R_c_* (1<*R_c_*<600 m) and of the 95% quantile (1<
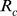
<600 m) of the Gaussian (left) and Cauchy (right) dispersal kernels. The dispersal dependent (DD) regime encompasses situations when *I˜* is mostly sensitive to 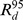 (vertical portion of the isolines), while the size dependent (SD) regime sets in when *I˜* is mostly sensitive to *R_c_* (horizontal portion of the isolines).

We review in Table 1 SINM estimates of the immigration parameter that have been published in the context of rainforest tree communities, either directly as *I* estimates or as *m* estimates (which were converted to *I* thanks to the above formula). We distinguished estimates that were based on the analysis of single large forest plots (10–50 ha) from estimates based on multiple smaller forest plots (*c.* 1 ha). These estimates were inferred using any of the four main published methods: (i) curve fitting of a SAD sensu [25], (ii) exact maximum-likelihood based on a coalescent approach (Etienne's sampling formula, ESF [7]) and variants [24], (iii) Approximate Bayesian Computation with simulations making use of the phylogenetic relationship between species within a field plot [23], or (iv) estimators based on similarity statistics (*G_ST_*(*k*) [5]).

### A kernel-based analogue of the neutral immigration number

We considered a kernel-based analogue (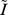
_)_ of the SINM immigration parameter, as a function of community radius, *R_c_*, and of a parameter of the dispersal kernel, say *R_d_*, such that 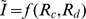. In this model, the local community was a disc of radius *R_c_* embedded into a spatially uniform, infinite two-dimensional landscape (Figure 1 right). The individuals from both the community and the neighbouring landscape were considered to be spread out at an average density *ρ* per unit area. Each individual dispersed the same number of propagules per generation following a specified dispersal kernel *q*(*r*), which was the probability density function of dispersal distances. The immigration parameter associated to the dispersal process, 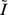, represents the number of immigrants competing with local offspring for the replacement of a dead individual in the community. The incoming migrants were modelled from the number of propagules falling within the boundaries of the community from an outside source, *N_out_*, while the local progeny were modelled from the number of propagules of residents that fall within the community, *N_in_*. 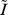 was thereby related to the ratio *N_out_*/*N_in_* to represent the relative number of immigrants competing for replacement, so that (Appendix S1 of [7]):
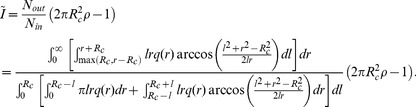
(1)



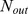
 and 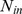 were obtained by summing over all sources located at distance *l* from the centre of the community. In the absence of a closed form expression for Eq. (1), we integrated it numerically (using Mathematica 8, Wolfram Research, Champaign, Illinois, USA) based on our given dispersal kernel functions *q*(*r*) (see below). For all subsequent calculations, we fixed the density *ρ* at 400 individuals per hectare, a value typically observed in tropical rainforests for trees with a minimal diameter at breast height above or equal to 10 cm diameter at breast height (dbh).

### Dispersal kernels

As advocated by previous authors [15], [26], [27], we assumed a rotational symmetry for *q*(*r*) and normalised the kernel density over 2D space accordingly:



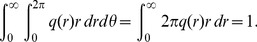
(2)


We thus examined two dispersal kernels traditionally used to fit seed distribution patterns from seed traps by inverse methods [26]. The first kernel defines an exponential family of curves [15]: 
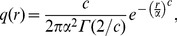
(3)which for shape parameter *c*  =  2 is the well-known Gaussian curve, and where *α* is a dispersal parameter and Γ the gamma function. A second kernel is the two dimensional Student’s *t* distribution, denoted as Student 2Dt [15], [26]–[28], which was used in [17] to model seed dispersal. However, we used a slightly modified version of the formula in [17] (see Eq. 5.1 of [29]), so that the dispersal parameter *u* could be expressed in standard units of distance (i.e. in m instead of m^2^ in the original formula). This dispersal kernel can be written as:
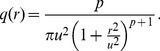
(4)


It corresponds to the heavily fat-tailed bivariate Cauchy kernel to model long distance dispersal (LDD) events [30], when the shape parameter *p*  =  0.5 [29]. With *p* becoming large, Eq. (4) reduces to a Gaussian dispersal kernel [17]. Note that our choice of the dispersal kernels is meant to address the relative influence of thin-tailed vs. fat-tailed kernels, which are known to affect diversity patterns in contrasted ways [31].

### Calculation of the dispersal parameter

Direct experimental information on seed dispersal distances is generally based on seed traps, thus results are often presented in terms of the median (the distance at which 50% of seeds fall before and 50% beyond) or maximal distance found. However the "maximal" distance, as observed in a given field study, may actually be overtaken by rare long dispersal events which are notoriously difficult to observe directly, and is therefore a statistic highly sensitive to the noisy and outlying variation [32]. In these cases, quantile statistics, which are generally more robust to such variation, can be used for dispersal kernel modelling [33]. In this paper, we equated the 95% quantile of dispersal distances in the dispersal kernel with the 'maximal' distance given from field observations (we also used the 90% quantile for testing the robustness of our results). We further considered the 50% quantile since some studies reported the median dispersal distance. Thus, we parameterized the dispersal kernels with the help of empirical dispersal distances which were considered as the 50% (median), 90% or 95% quantiles. For a Student’s 2Dt dispersal kernel, the fraction of seeds dispersed beyond the radius *R* is 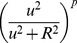, which can be written as 
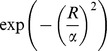
 in the Gaussian limit when *p* goes to infinity ([15]: 1489). We then calculated *u* (for Cauchy with *p*  =  0.5) and *α* (for the Gaussian limit) so that this fraction was 50% (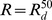), 10% (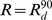) or 5% (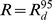). Specifically, in the Gaussian limit,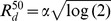, while in the Cauchy case, 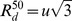.

Here the kernel-based 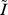 is calculated under the assumption that dispersal limitation is the only component of limited migration found in real communities. Other extrinsic barriers to migration, including physical and ecological barriers, can still decrease the immigration fluxes below this baseline value. Thus, a comparison of the spatially-implicit and kernel-based immigration parameters allows assessing the contribution of each of these factors.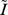 was therefore evaluated numerically for both dispersal kernels, and compared to published values of *I* estimated from tropical forests plots, under variants of the SINM. We calculated *R_c_* for a disk of same area than the plot, and deduced 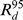 (as well as 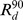 and 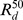) that would allow 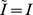 (Table 1 and Table S1). We further compared the values of 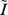 with those based on the approximate formula of Chisholm and Lichstein [17] which illustrated, within the limit of their approximation, the validity of our approach (Appendix S1).

## Results

### Kernel-based predictions of the migration-drift equilibrium

Figure 2 presents contour lines of 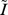 obtained for the two dispersal kernels by varying the community radius *R_c_* and the dispersal parameter (95% quantile). Computations were conducted to represent a range of community radii from 1 to 600 m (i.e. a disk area from 3×10^−4^ to 113 ha) which largely includes the range of forest plot sizes presented in Table 1. Also, the range of dispersal distances from 1 to 600 m encompasses the largest values observed in tropical forests as reported in Table 2 and Figure S1. Our study of 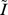 found two contrasted situations: in the case of the Cauchy kernel, 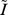
was mainly sensitive to variation in community size, *R_c_* (horizontal isolines in Figure 2 right), except for very small 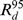, while in the case of the Gaussian kernel, 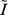 was mainly sensitive to variation in the dispersal distances, as embodied by (vertical isolines in Figure 2 left). We denote the former situation as a ‘size dependent’ (SD) regime, because in this case the outcome of the migration-drift dynamics on community composition depends far more on a change in the size of the community (which determines the effect of drift) than on a change in the dispersal parameter 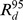. Conversely we call the second situation a ‘dispersal dependent’ (DD) regime, because in this case the migration-drift isolines are strongly sensitive to variation in the dispersal parameter. Although the isolines of 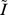 were established with respect to the same realistic ranges of *R_c_* and from published data, they varied from 0 to more than 10000 in the Gaussian case, but only from 0 to a little above 100 in the Cauchy case (Figure 2). Thus the nature of the dispersal kernel strongly influenced the expected migration-drift equilibrium experienced by the community.

**Figure 3:**
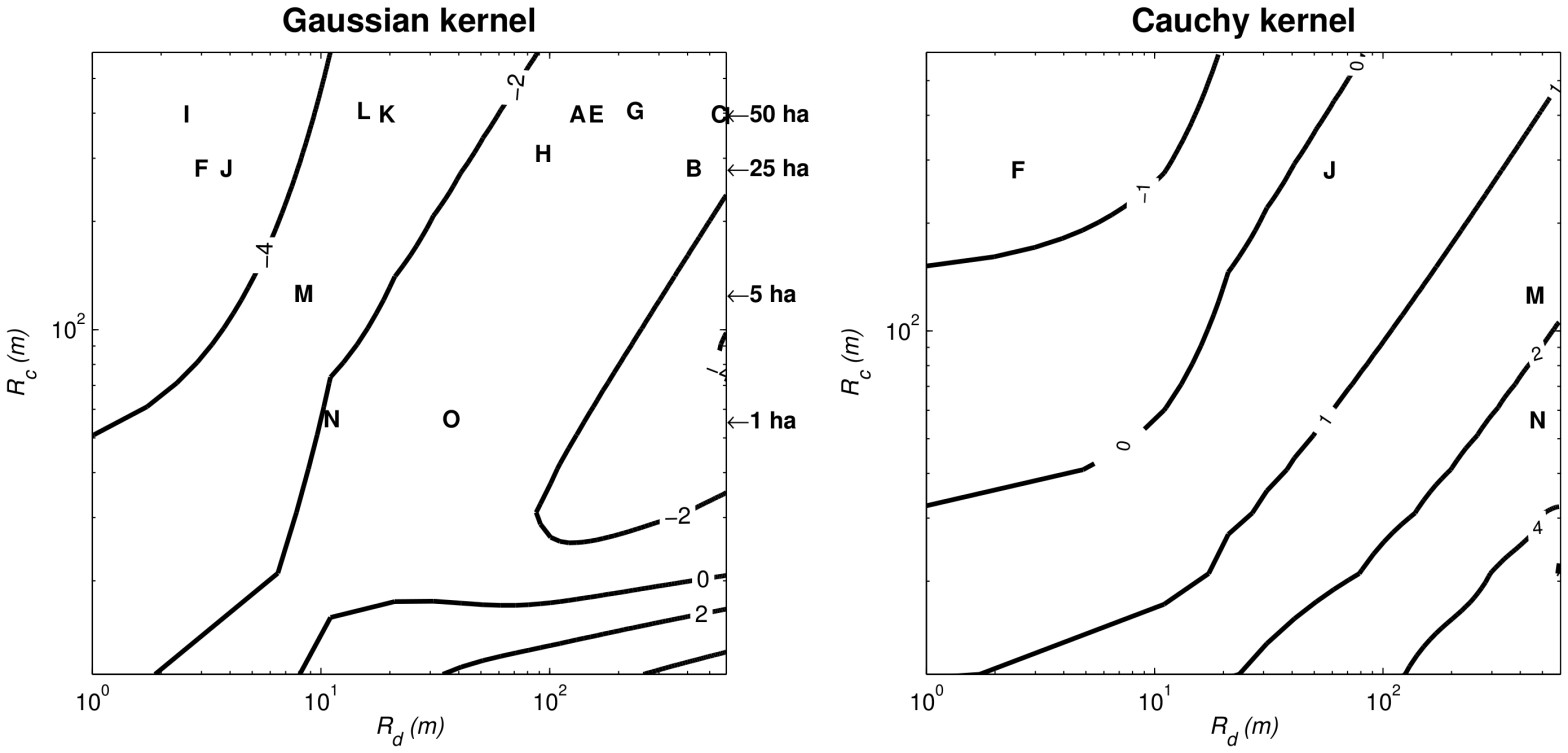
Sensitivity of the immigration parameter *I˜*, as predicted from Gaussian and Cauchy dispersal kernels, to dispersal distance and local community radius. Isolines of 
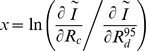
 are functions of the community radius *R_c_* (10<*R_c_* <600 m) and of the 95% quantile, 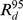 (1<
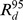
<600 m), for the Gaussian (left) and Cauchy (right) dispersal kernels. The null isoline of *x* represents the limit (equal sensitivity to the variation in *R_c_* and 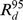) between the dispersal dependent (DD, *x*<0) and size dependent (SD, *x* > 0) zones (see main text). We displayed field plot locations (using the codes in Table 1) by calculating the 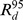 values corresponding to each plot size. Most of the plots fall into the DD and SD zones for the Gaussian Cauchy kernel, respectively.

The sensitivity of 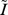 to the changeover between the SD and the DD regimes was also studied using the log-ratio of the partial derivatives of 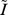 with respect to *R_c_* and (Figure 3). This ratio cancels out when variations in *R_c_* and contribute equally to the net change of 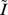. It becomes positive under the SD regime and negative under the DD regime. Note that the sensitivity of the migration-drift equilibrium to a change in *R_c_* and is independent of community density (which vanishes in the ratio). Figure 3 (left) illustrates the change of regime for the Gaussian kernel, which occurs for a community radius of 10 to 20 m (less than 0.12 ha). Similarly, Figure 3 (right) reveals that the Cauchy kernel is mainly associated with a size dependent situation (positive log-ratio).

### Comparison with inferred values and empirical dispersal distances

Table 1 shows that *I* values previously derived from species abundance data for a single large plot range from 1195.3 to 29693, except for the Sinharaja plot (32.3). Most of them are thus several orders of magnitude above the values deduced either from networks of smaller plots, which do not exceed 171, or from methods acknowledging the community phylogenetic structure of a single large plot [23], which do not exceed 300. Moreover, substantial variation can be found between estimates from surveys even within the same forest. For instance, regarding the Barro Colorado Island plot, there was considerable variation between the initial assessment [7] and the values found in later works [17], [23], [34].

 We further addressed to what extent the published estimates of *I* in Table 1 could be interpreted as resulting from a dispersal process as modelled via kernel functions (Eq. 1). We assumed that plot sizes equalled community sizes to calculate *R_c_*, then computed for the *I* estimates of Table 1 the (resp., 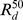 and 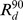) values that allowed *I˜* to match *I*, and mentioned their corresponding positions on Figure 3. The values of (resp., 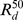 and 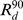) are included in Table 1 (resp., Table S1). Note that Figure 3 is independent of the density of individuals found among the tropical plots, which allows us to plot the various expected values as long as they are within a realistic range of dispersal distances. Only four estimates of *I* in Table 1 could be associated with a range of realistic in the Cauchy case (all other plots were outside Figure 3 right) while most of the plots were present within this range for the Gaussian kernel (Figure 3 left), except Korup (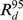 =  818 m). For the Cauchy kernel, the derived was often above 10 km (Table 1), which is clearly unrealistic for any kind of dispersal mode. The results were qualitatively consistent when considering 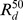, or when using 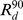 instead of as a proxy of long-dispersal distance (Table S1).

A focal community of 1 ha (*R_c_* ≈ 56 m), would always correspond to a DD situation in the Gaussian case and to a SD regime for the Cauchy case. For *R_c_*  =  56 m and according to a Gaussian kernel, letting range from 10 to 60 m results in *I˜* values between 24 and 204 which is consistent with the *I* estimated for networks of 1 ha plots in Table 1. This exemplifies the notion of a DD regime since most of the variation of *I˜* can be explained by variations of within the range of realistic dispersal distances for tropical rainforests although zoochorous dispersal can yield a still higher (Table 2 and Figure S1). For the Cauchy case (Figure 2 right), an *I˜* > 60 at *R_c_*  =  56 m requires a very high (2831 m), which is clearly unrealistic, even in the case of zoochory (Table 2 and Figure S1). In the Gaussian case, we further note that attaining *I˜* values in the range of 2000–5000 (i.e., roughly the range of estimates from large plots in Table 1) with kept at 200 m (compatible with zoochory) requires 145 m<*R_c_*<538 m (6.61 ha<*R_c_*<90.93 ha). With the Cauchy kernel, a similar variation in *R_c_* yields 32<*I˜*< 89 (with kept at 200 m), while values substantially above 100 are unattainable for any meaningful value of *R_c_*. Finally, some estimates like those of Korup (i.e. *I* > 13000, see Table 1) are definitely too high, whichever the kernel used (plot outside Figure 3).

## Discussion

In his spatially-implicit neutral model (SINM), Hubbell promoted migration as the central driver of local community diversity and assembly whilst speciation processes operate over the long term at regional scale [2]. Subsequent works have questioned this spatially-implicit formulation [22] and pursued a complementary line of research that focuses on the effects of propagule dispersal on population dynamics and community assembly, with particular interest into the role of long-distance dispersal [28], [35]–[37]. These studies, which model variations in dispersal characteristics using kernel functions, further instigated research into the reconciliation between the concept of spatially-implicit migration limitation and the kernel-based formulation of pure dispersal limitation around parent individuals [7], [17], [38], [39]. In this paper, we provide novel insights into this issue. In the context of tropical forest communities, we compiled published values of the SINM immigration parameter *I* estimated from field plots, along with observations of seed dispersal distances. Secondly, we derived predictions of the immigration parameter alternatively using two contrasting dispersal kernels (i.e., Gaussian vs. Cauchy). By exploring a large range of plausible dispersal distances and community sizes (with respect to field data) we checked for inconsistencies between the kernel-based prediction (denoted as *I˜*) and the spatially-implicit estimates (i.e. *I*) inferred from tropical field plots (Table 1).

Our study highlights that the kernel-predicted immigration parameter *I˜* is liable to switch between two extreme states (Figures 2 and 3), respectively shaped by size dependence (or SD) where it is more sensitive to changes in community size, and dispersal dependence (or DD) where it is primarily sensitive to changes in dispersal distances. Such a result is consistent with the definition of *I* as a migration-drift parameter [2]. The heavily fat-tailed Cauchy kernel and the Gaussian kernel thus correspond to SD and DD regimes, respectively. Fat-tailed dispersal kernels from outside sources are flatter over the local community area and thus changing dispersal distance does not make much difference to that flatness, whereas Gaussian dispersal kernels decay more sharply in the tails and thus dispersal distance has more effect when integrated over the local community. Kernel-based modelling can thus be used to assess whether variations in *I* values inferred from real communities may be due to either variations in community size or dispersal distances. Furthermore, we can expect actual *I* values to be lower than the predicted *I˜* (i.e. under strict propagule dispersal) when ecological factors reinforce the isolation of local communities (e.g. physical or anthropogenic barriers) by restricting immigration. Conversely, *I* values larger that *I˜* (greater immigration than expected under strict dispersal limitation) are difficult to explain and probably highlight estimation problems such as the violation of some of the main assumptions of the SINM-derived estimation methods.

The considerable variation in published *I* values in Table 1 is conspicuously related to the size of the field forest plots in which species abundance information was recorded. Very large values of *I*, which are associated with single large plots, cannot be consistent with a fat-tailed dispersal kernel (Cauchy kernel, Figure 2 right), unless the reference dispersal range (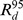, i.e., the quantile at 95%) is unrealistically large (above 10 km). However a Gaussian dispersal kernel agreed with such values, insofar as is approximately above 100 m and below 500 m, which is mainly consistent with data on zoochory (Table 2 and Figure S1). For fixed 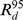, larger *I* values can of course be found by increasing community size, but very large communities are expected to violate the SINM’s fundamental assumption of panmixia within communities, which posits that all individuals in the community have the same probability of contributing a descendant to the replacement of a dead individual. A large plot could then contain several distinct communities and therefore appear globally more diverse than each community taken alone. Besides, large plots are also subject to habitat heterogeneity, as reported in several of the large plots mentioned in Table 1 (e.g., [40], [41]). Finally, under the assumption of panmixia within local communities, coalescent-based models of community dynamics assume that *I*, contrary to *m,* is independent of the size of a sample plot embedded in the reference community (Figure 1 left). Accordingly, the broad variation that is observed among the estimates of *I* within comparable forest communities (cf. Table 1 for plots of different sizes within the Barro Colorado area) should be seen as an anomaly. We therefore advocate the use of many smaller samples, whose estimates are probably more reliable, as they are less likely to violate the panmixia assumption of the SINM.

The small *I* estimates in Table 1 are obtained through very different estimation techniques (e.g., [5] vs. [24]), appear much less variable and are probably more ecologically relevant than their larger counterparts. Moreover, values similar to those estimated from multiple plot datasets were found when single-plot estimates accounted for the phylogenetic relationship between tree species [23]. Studies inferring *I* from a network of forest plots are unfortunately rare and limited to the regions mentioned in Table 1, but the upper range of estimated *I* values (i.e. 80-150) easily match with *I˜* in the Gaussian case for realistic dispersal distances (Figure 2). However, smallest values (10<*I*<20) imply very small dispersal distances for the case of a Gaussian kernel (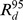
<10 m, Fig. 2 left). The same variation of *I* estimates is possible under a wider range of dispersal distances for the heavily fat-tailed Cauchy, but this would mean that the community is smaller than the reference plot (*R_c_*<1 ha, Fig. 2, right). Therefore, in order to explain such low estimates one has to either assume very low dispersal distances or very small communities. The first case would mean that dispersal modes of low range (e.g. barochory) are dominant though the available literature seems to present zoochory as the most frequent mode in tropical rainforests (cf. Table 2 and Figure S1). The second case begs the question as to whether the widely used 1-ha plots are a reasonable limit. Other explanations imply that strict dispersal limitation alone, as modelled by a kernel function, cannot account for the low observed values and the reported *I* values may therefore encompass various sources of migration limitation. If so, one should not interpret *I* values as the outcome of a pure dispersal limitation model. Also, the tree communities, usually sampled at a 10 cm limit for the diameter at breast height (dbh), may not acknowledge all the reproductive trees and may underestimate local diversity, which would tend to decrease *I* estimates. Finally, our results illustrate the relevance of extending the scope of the neutral models to better acknowledge other sources of migration limitation from the regional background, by habitat filtering or biogeographical barriers (see [24]).

Apart from these awaited and ongoing developments, we can nevertheless underline the usefulness of the SINM framework which provides, via the immigration parameter *I,* a phenomenological measure of community isolation from its regional biogeographic background. The use and interpretation of *I* as such does not require any assumption on the nature of the migration limitation (ex. pure dispersal limitation). Neither does it require any assumption that local communities are part of a panmictic metacommunity at speciation-drift equilibrium [5], thereby enabling the estimation of *I* independently from any assumption on the process of speciation. This property renders the SINM, or at least some of its variants, highly complementary to the spatially explicit formulation under which dispersal and speciation have been depicted so far with intertwined roles [29]. Thus, the difficult question of the theoretical relationship between spatially-implicit and -explicit neutral models may be translated into the more pragmatic prospect of analyzing how the SINM-based estimation of immigration is sensitive to local and regional causes of community isolation. Interpreting *I* as an isolation index then only requires the assumption that the available data are sufficiently informative about the taxonomic composition of the regional background around the focal community (species pool). The design of species pools has recently become a major challenge of community ecology [42], [43], and the linking of this issue to that of assessing migration processes from regional to local scales opens promising perspectives. Similar approaches could contribute to comparisons between continents and regions in terms of individual and/or species mobility and help bridge the gap between the community and biogeographic scales.

## Supporting Information

Figure S1 **Frequency distribution of the mean dispersal distances of 260 rainforest tree species from French Guiana, categorized into dispersal modes**. Data compiled from the online data of species traits http://mariwenn.ecofog.gf/ [19].
(DOC)

Table S1
**The 50% and 90% quantile dispersal distances,** 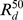 **and** 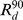 **, corresponding to the rainforest field plots of** **Table 1**. The 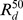 and 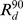 values are provided for the purpose of comparison with the 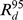 values given in the main text (Table 1). The *I* values are published estimates of the immigration parameter using Spatially Implicit Neutral Models (SINMs). *A* (sample area in ha) corresponds to *J_s_* (the plot sample size in number of individuals) assuming a density 400 individuals/ha of trees above 10 cm dbh. 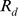 values have been rounded to the nearest metre.(DOC)

Appendix S1 **Rewriting Chisholm and Lichstein’s analytical expression.**
(DOC)
