# Supplementary figures and images for: Do Spatially-Implicit Estimates of Neutral Migration Comply with Seed Dispersal Data in Tropical Forests?
Source: PLoS One. 2013 Aug 19;8(8):e72497. doi: 10.1371/journal.pone.0072497 (PMC3747097; doi:10.1371/journal.pone.0072497)

**Figure S1**


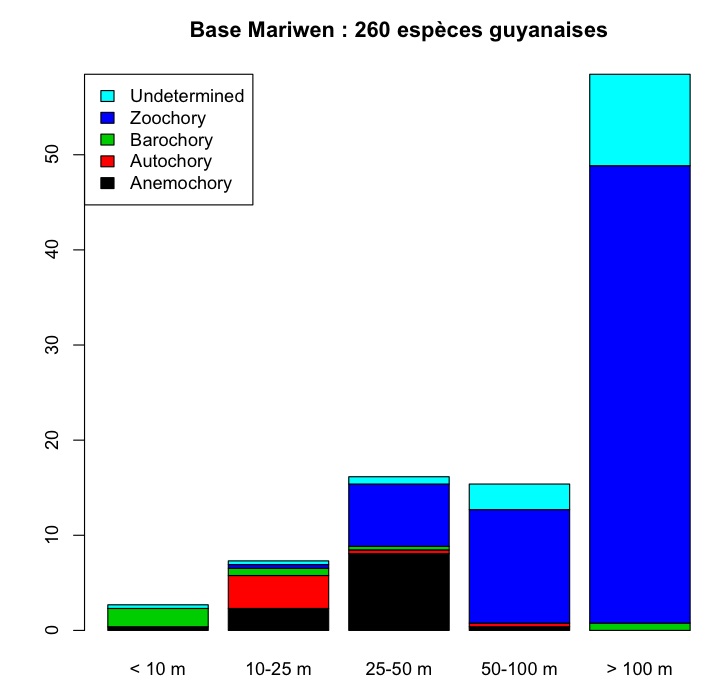

Supplement: Figure S1 — Frequency distribution of the mean dispersal distances of 260 rainforest tree species from French Guiana, categorized into dispersal modes. Data compiled from the online data of species traits http://mariwenn.ecofog.gf/ [19]. (DOC) [file pone.0072497.s001.doc]
