# Supplementary material for: Do Spatially-Implicit Estimates of Neutral Migration Comply with Seed Dispersal Data in Tropical Forests?
Source: PLoS One. 2013 Aug 19;8(8):e72497. doi: 10.1371/journal.pone.0072497 (PMC3747097; doi:10.1371/journal.pone.0072497)
Supplement: Table S1 — The 50% and 90% quantile dispersal distances, and , corresponding to the rainforest field plots of Table 1 . The and values are provided for the purpose of comparison with the values given in the main text (Table 1). The I values are published estimates of the immigration parameter using Spatially Implicit Neutral Models (SINMs). A (sample area in ha) corresponds to Js (the plot sample size in number of individuals) assuming a density 400 individuals/ha of trees above 10 cm dbh. values have been rounded to the nearest metre. (DOC) [file pone.0072497.s002.doc]

|  |  | *A* | *J_s_* | *I* | $R_{d}^{50}(Gaussian)$ | $R_{d}^{50}(Cauchy)$ | $R_{d}^{90}(Gaussian)$ | $R_{d}^{90}(Cauchy)$ |
| --- | --- | --- | --- | --- | --- | --- | --- | --- |
|  | **Single large forest plot datasets** |  |  |  |  |  |  |  |
|  | *Based on taxonomic diversity alone* |  |  |  |  |  |  |  |
| A | Barro Colorado Island, Panama [2] | 50 ha | 21457 | 2200 | 58 m | 4805 m | 107 m | >10 km |
| B  C | Yasuni National Park, Ecuador [2,3] | 25 ha 50 ha | 7613  17546 | 7612  13182 | 188 m  242 m | >10 km  >10 km | 343 m  442 m | >10 km  >10 km |
| D | Korup National Park, Cameroon [2] | 50 ha | 24591 | 29693 | 393 m | >10 km | 717 m | >10 km |
| E | Pasoh Forest Reserve, Malaysia [2] | 50 ha | 26554 | 2722.6 | 71 m | 5476 m | 129 m | >10 km |
| F | Sinharaja, Sri Lanka [2] | 25 ha | 16936 | 32.3 | 1 m | <1 m | 2 m | 1 m |
| G | Lambir Hills, Malaysia [2] | 52 ha | 33175 | 4310.7 | 104 m | 7364 m | 189 m | >10 km |
| H | Western Ghats, India [4] | 30 ha | 13383 | 1195.3 | 41 m | 3038 m | 75 m | >10 km |
|  | *Including phylogenetic information* [5] |  |  |  |  |  |  |  |
| I | Barro Colorado Island, Panama | 50 ha | 20788 | 41.66 | 1 m | ~0 | 2 m | ~0 |
| J | La Planada, Colombia | 25 ha | 14100 | 42.42 | 2 m | 5 m | 3 m | 27 m |
| K | Pasoh Forest Reserve, Malaysia | 50 ha | 29257 | 295.5 | 9 m | 946 m | 16 m | 5437 m |
| L | Lambir Hills Forest Dynamics Plot, Malaysia | 52 ha | 29890 | 241.0 | 7 m | 538 m | 13 m | 3091 m |
|  | **Multiple plot datasets** |  |  |  |  |  |  |  |
| M | Baro Colarado subplot, Cocoli and Sherman plots, Panama [6] | ~5 ha/plot | 1079-2860 | 30.7-54.2 | 4 m | 36 m | 7 m | 209 m |
| N | Western Ghats, India [7] | 1 ha/plot | ~400/plt | 4.7-50; 1.3-35^4^ | 5 m | 38 m | 9 m | 217 m |
| O | Panama Canal Watershed [8] | 1 ha/plot | ~400/plt | 21-171 | 16 m | 376 m | 30 m | 2159 m |
