# Supplementary material for: Do Spatially-Implicit Estimates of Neutral Migration Comply with Seed Dispersal Data in Tropical Forests?
Source: PLoS One. 2013 Aug 19;8(8):e72497. doi: 10.1371/journal.pone.0072497 (PMC3747097; doi:10.1371/journal.pone.0072497)
Supplement: Appendix S1 — Rewriting Chisholm and Lichstein’s analytical expression. (DOC) [file pone.0072497.s003.doc]

**Appendix S1. Rewriting Chisholm and Lichstein’s analytical expression**

Chisholm and Lichstein (Appendix S5 in [1]) proposed an approximate analytical expression of the kernel-based immigration parameter $\tilde{I}$for the case when the corresponding migration rate $\tilde{m}$ << 1 and the mean dispersal distance *d* << *P*, where *P* is the perimeter of the community. We compared to the exact $\tilde{I}$ for the Gaussian dispersal kernel. In this case, the mean dispersal distance is $d=\alpha\sqrt{\pi}/2=R_{d}^{50}\sqrt{\frac{\pi}{4\log(2)}}$ ($R_{d}^{50}$ is the median dispersal distance). In the case of circular communities of radius *R_c_*, we further translated *P* into 2π*R_c_*, so that

(Eq. A1)

We could not calculate *I_C&L_* for the Cauchy dispersal kernel, because in this case mean dispersal distance remains undefined [2].

In Figure A1 below, we plotted the “error” between $\tilde{I}$ and *I_C&L_* for systematic combinations of plausible community size *R_c_* and median dispersal distance $R_{d}^{50}$. We found that both the quantities agreed very well for the range of conditions considered in [1], i.e. for a Gaussian dispersal kernel with small median dispersal distance and large community size (i.e., $w=R_{d}^{50}/R_{c}<<1$). $\tilde{I}$ and *I_C&L_* however diverged when the median dispersal distance got large relatively to community size (i.e. when *w* got larger than 1). These results illustrate to what extent *I_C&L_* becomes biased with regard to $\tilde{I}$ outside its domain of validity.

**Figure A1.** A comparison of the two kernel-based estimates of the immigration parameter, i.e. our $\tilde{I}$and *I_C&L_* of Chisholm and Lichstein (see Methods, Eq. 1, and present Appendix, Eq.A1), for a Gaussian seed dispersal kernel. The community radius *R_c_* and the median dispersal distance $R_{d}^{50}$ vary within [3m ; 600m]. The *y*-axis represents the relative error between $\tilde{I}$and *I_C&L_*, while the *x*-axis is given in terms of the ratio $w=R_{d}^{50}/R_{c}$.

**References linked to Supporting information**

1. Chisholm RA, Lichstein JW (2009) Linking dispersal, immigration and scale in the neutral theory of biodiversity. Ecol Lett 12: 1385–1393.

2. Clark JS, Silman M, Kern R, Macklin E, HilleRisLambers J (1999) Seed Dispersal Near and Far: Patterns Across Temperate and Tropical Forests. Ecology 80: 1475-1494.
